# Supplementary material for: Pyruvate Dehydrogenase Kinase 1 inhibition mediated oxidative phosphorylation enhancement in cartilage promotes osteoarthritis progression
Source: BMC Musculoskelet Disord. 2023 Jul 20;24:597. doi: 10.1186/s12891-023-06585-6 (PMC10357736; doi:10.1186/s12891-023-06585-6)
Supplement: Supplementary file 1 — Additional file 1. [file 12891_2023_6585_MOESM1_ESM.zip › MF.Gsea.1653627316242/GOMF_PEPTIDE_N_ACETYLTRANSFERASE_ACTIVITY.html]

Details for gene set GOMF\_PEPTIDE\_N\_ACETYLTRANSFERASE\_ACTIVITY[GSEA]

|  || Dataset | 55457\_collapsed\_to\_symbols.cls.cls #OA\_versus\_normal.cls.cls #OA\_versus\_normal\_repos |
| Phenotype | cls.cls#OA\_versus\_normal\_repos |
| Upregulated in class | normal |
| GeneSet | GOMF\_PEPTIDE\_N\_ACETYLTRANSFERASE\_ACTIVITY |
| Enrichment Score (ES) | -0.40843594 |
| Normalized Enrichment Score (NES) | -1.4635708 |
| Nominal p-value | 0.045150504 |
| FDR q-value | 0.21699095 |
| FWER p-Value | 1.0 |
Table: GSEA Results Summary

  

Fig 1: Enrichment plot: GOMF\_PEPTIDE\_N\_ACETYLTRANSFERASE\_ACTIVITY      
 Profile of the Running ES Score & Positions of GeneSet Members on the Rank Ordered List

  

| SYMBOL | TITLE | RANK IN GENE LIST | RANK METRIC SCORE | RUNNING ES | CORE ENRICHMENT || 1 | KAT8 | lysine acetyltransferase 8 [Source:HGNC Symbol;Acc:HGNC:17933] | 595 | 0.625 | -0.0002 | No |
| 2 | KAT7 | lysine acetyltransferase 7 [Source:HGNC Symbol;Acc:HGNC:17016] | 1125 | 0.491 | -0.0051 | No |
| 3 | GTF2B | general transcription factor IIB [Source:HGNC Symbol;Acc:HGNC:4648] | 1315 | 0.455 | 0.0137 | No |
| 4 | ARRB1 | arrestin beta 1 [Source:HGNC Symbol;Acc:HGNC:711] | 2116 | 0.348 | -0.0229 | No |
| 5 | HAT1 | histone acetyltransferase 1 [Source:HGNC Symbol;Acc:HGNC:4821] | 2118 | 0.348 | 0.0027 | No |
| 6 | NAA60 | "N-alpha-acetyltransferase 60, NatF catalytic subunit [Source:HGNC Symbol;Acc:HGNC:25875]" | 2489 | 0.303 | -0.0038 | No |
| 7 | KAT6B | lysine acetyltransferase 6B [Source:HGNC Symbol;Acc:HGNC:17582] | 2827 | 0.267 | -0.0103 | No |
| 8 | CLOCK | clock circadian regulator [Source:HGNC Symbol;Acc:HGNC:2082] | 3231 | 0.227 | -0.0249 | No |
| 9 | MED24 | mediator complex subunit 24 [Source:HGNC Symbol;Acc:HGNC:22963] | 3246 | 0.226 | -0.0093 | No |
| 10 | NAA10 | "N-alpha-acetyltransferase 10, NatA catalytic subunit [Source:HGNC Symbol;Acc:HGNC:18704]" | 3477 | 0.205 | -0.0121 | No |
| 11 | NAA11 | "N-alpha-acetyltransferase 11, NatA catalytic subunit [Source:HGNC Symbol;Acc:HGNC:28125]" | 3620 | 0.192 | -0.0090 | No |
| 12 | BRCA2 | BRCA2 DNA repair associated [Source:HGNC Symbol;Acc:HGNC:1101] | 3853 | 0.170 | -0.0145 | No |
| 13 | USP22 | ubiquitin specific peptidase 22 [Source:HGNC Symbol;Acc:HGNC:12621] | 4687 | 0.097 | -0.0721 | No |
| 14 | KAT2B | lysine acetyltransferase 2B [Source:HGNC Symbol;Acc:HGNC:8638] | 4867 | 0.082 | -0.0799 | No |
| 15 | MSL3 | MSL complex subunit 3 [Source:HGNC Symbol;Acc:HGNC:7370] | 5596 | 0.024 | -0.1348 | No |
| 16 | KAT6A | lysine acetyltransferase 6A [Source:HGNC Symbol;Acc:HGNC:13013] | 5778 | 0.010 | -0.1481 | No |
| 17 | TAF9 | TATA-box binding protein associated factor 9 [Source:HGNC Symbol;Acc:HGNC:11542] | 6297 | -0.027 | -0.1864 | No |
| 18 | KAT5 | lysine acetyltransferase 5 [Source:HGNC Symbol;Acc:HGNC:5275] | 6379 | -0.034 | -0.1902 | No |
| 19 | MCM3AP | minichromosome maintenance complex component 3 associated protein [Source:HGNC Symbol;Acc:HGNC:6946] | 8077 | -0.161 | -0.3102 | No |
| 20 | CDY1 | chromodomain Y-linked 1 [Source:HGNC Symbol;Acc:HGNC:1809] | 8096 | -0.163 | -0.2996 | No |
| 21 | NAA80 | "N-alpha-acetyltransferase 80, NatH catalytic subunit [Source:HGNC Symbol;Acc:HGNC:30252]" | 8328 | -0.180 | -0.3043 | No |
| 22 | ATF2 | activating transcription factor 2 [Source:HGNC Symbol;Acc:HGNC:784] | 8592 | -0.201 | -0.3100 | No |
| 23 | NAA40 | "N-alpha-acetyltransferase 40, NatD catalytic subunit [Source:HGNC Symbol;Acc:HGNC:25845]" | 8791 | -0.216 | -0.3095 | No |
| 24 | NCOA1 | nuclear receptor coactivator 1 [Source:HGNC Symbol;Acc:HGNC:7668] | 9270 | -0.253 | -0.3280 | No |
| 25 | ATAT1 | alpha tubulin acetyltransferase 1 [Source:HGNC Symbol;Acc:HGNC:21186] | 9329 | -0.258 | -0.3136 | No |
| 26 | KAT2A | lysine acetyltransferase 2A [Source:HGNC Symbol;Acc:HGNC:4201] | 9799 | -0.301 | -0.3278 | No |
| 27 | NAA50 | "N-alpha-acetyltransferase 50, NatE catalytic subunit [Source:HGNC Symbol;Acc:HGNC:29533]" | 10044 | -0.326 | -0.3228 | No |
| 28 | CREBBP | CREB binding protein [Source:HGNC Symbol;Acc:HGNC:2348] | 10881 | -0.414 | -0.3573 | No |
| 29 | SUPT7L | "SPT7 like, STAGA complex subunit gamma [Source:HGNC Symbol;Acc:HGNC:30632]" | 11540 | -0.506 | -0.3712 | Yes |
| 30 | GTF3C4 | general transcription factor IIIC subunit 4 [Source:HGNC Symbol;Acc:HGNC:4667] | 11621 | -0.520 | -0.3391 | Yes |
| 31 | EP300 | E1A binding protein p300 [Source:HGNC Symbol;Acc:HGNC:3373] | 11741 | -0.540 | -0.3086 | Yes |
| 32 | NAA16 | "N-alpha-acetyltransferase 16, NatA auxiliary subunit [Source:HGNC Symbol;Acc:HGNC:26164]" | 11813 | -0.552 | -0.2734 | Yes |
| 33 | TADA2A | transcriptional adaptor 2A [Source:HGNC Symbol;Acc:HGNC:11531] | 12217 | -0.650 | -0.2569 | Yes |
| 34 | BAZ1A | bromodomain adjacent to zinc finger domain 1A [Source:HGNC Symbol;Acc:HGNC:960] | 12238 | -0.655 | -0.2102 | Yes |
| 35 | NCOA3 | nuclear receptor coactivator 3 [Source:HGNC Symbol;Acc:HGNC:7670] | 12455 | -0.727 | -0.1734 | Yes |
| 36 | SRCAP | Snf2 related CREBBP activator protein [Source:HGNC Symbol;Acc:HGNC:16974] | 12542 | -0.769 | -0.1234 | Yes |
| 37 | NAA15 | "N-alpha-acetyltransferase 15, NatA auxiliary subunit [Source:HGNC Symbol;Acc:HGNC:30782]" | 12693 | -0.859 | -0.0718 | Yes |
| 38 | TAF1 | TATA-box binding protein associated factor 1 [Source:HGNC Symbol;Acc:HGNC:11535] | 12881 | -1.192 | 0.0015 | Yes |
Table: GSEA details [plain text format]

  

Fig 2: GOMF\_PEPTIDE\_N\_ACETYLTRANSFERASE\_ACTIVITY      
 Blue-Pink O' Gram in the Space of the Analyzed GeneSet

  

Fig 3: GOMF\_PEPTIDE\_N\_ACETYLTRANSFERASE\_ACTIVITY: Random ES distribution      
 Gene set null distribution of ES for **GOMF\_PEPTIDE\_N\_ACETYLTRANSFERASE\_ACTIVITY**
